# Overlapping patterns of neural activity for different forms of novelty in fMRI

**DOI:** 10.3389/fnhum.2014.00699

**Published:** 2014-09-08

**Authors:** Colin Hawco, Martin Lepage

**Affiliations:** ^1^Temerty Center for Therapeutic Brain Stimulation, Centre for Addiction and Mental Health, University of TorontoToronto, ON, Canada; ^2^Douglas Mental Health University Institute, McGill UniversityMontreal, QC, Canada

**Keywords:** repetition suppression, novelty, fMRI, semantic, priming

## Abstract

When stimuli are presented multiple times, the neural response to repeated stimuli is reduced relative to novel stimuli (repetition suppression). Responses to different types of novelty were examined. Stimulus novelty was examined by contrasting first vs. second presentation of triads of objects during memory encoding. Semantic novelty was contrasted by comparing unrelated (semantically novel) triads of objects to triads in which all three objects were related (e.g., all objects were tools). In recognition, associative novelty was examined by contrasting rearranged triads (previously seen objects in a new association) with intact triads. Activity was observed in posterior regions (occipital and fusiform), with the largest extent of activity for stimulus novelty and smallest for associational novelty. Frontal activity was also observed in stimulus and semantic novelty. Additional analysis indicated that the hemodynamic response in voxels identified in the stimulus and semantic novelty contrasts was modulated by reaction time on a trial-by-trial basis. That is, the duration of the hemodynamic response was driven by reaction time. This was not the case for associative novelty. The high level of overlap across different forms of novelty suggests a similar mechanism for reduced neural activity, which may be related to reduced visual processing time. This is consistent with a facilitation model of repetition suppression, which posits a reduced peak and duration of neuronal firing for repeated stimuli.

## Introduction

Repetition suppression is a widely observed phenomenon in which a decreased neural response is observed following a repeated stimulus compared to a novel stimulus. This phenomenon has been observed in single cell recordings in animals (Li et al., 1993) and at the level or large scale neural populations in humans using functional neuroimaging (Kirchhoff et al., 2000; Grill-Spector et al., 2006; Pihlajamaki et al., 2008; Poppenk et al., 2010; Xue et al., 2010). Larger blood oxygen level dependant (BOLD) signal in fMRI for novel compared to repeated stimulus (repetition suppression) has been observed in a wide variety of stimuli, including faces (Xue et al., 2010) and name/face pairs (Pihlajamaki et al., 2008), pictures or words (Kirchhoff et al., 2000), and scenes (Poppenk et al., 2010). While a number of different regions have demonstrated repetition suppression, the most consistently activated regions include the posterior cortex (occipital, inferior parietal and fusiform), although fairly consistent activity has also been noted in frontal cortex and the medial temporal lobes.

Several models have been posited to explain repetition suppression (Grill-Spector et al., 2006). Under the fatigue model, there is a decrease in overall firing rates of neurons following repeated stimuli, without a decrease in the number of neurons or duration of firing (Grill-Spector and Malach, 2001). The sharpening model proposes a process in which fewer neurons are responding to the repeated stimulus resulting in a decreased (“sharpened”) neural response (Desimone, 1996; Wiggs and Martin, 1998). Facilitation models proposes that object priming shortens the temporal peak of neural activity resulting in a shorter duration of neuronal firing (James and Gauthier, 2006), which will reduce the magnitude of the BOLD signal in fMRI and may decrease reaction times. While repetition suppression is often examined in short time scales (examining suppression to the presentation of the same stimulus twice in a row, (Grill-Spector et al., 1999), which produces the largest suppression response, (Henson et al., 2000; Sayres and Grill-Spector, 2006), it has also been observed across time scales of minutes (Henson et al., 2000) or even days (van Turennout et al., 2000). Repetition suppression can be thought of as either reduced neural activity to repeated stimuli, or increased neural activity to novel stimuli.

We conducted a memory encoding and recognition fMRI study for objects triads. Part of that study involving semantic processing was published earlier (Hawco et al., 2013). While this paradigm was designed in order to assess self-initiation of elaborative encoding, stimuli (triads of objects) were repeated twice during encoding and could be semantically related or unrelated, and a subsequent recognition test presented intact vs. rearranged combinations of previously seen stimuli. This allowed for a novel subsequent analysis focused on different forms of novelty which were embedded with the existing paradigm. Different types of “novelty” effects during encoding and recognition were considered. Stimulus novelty was examined by contrasting the first vs. second presentation of a group of objects, as is done in studies examining repetition suppression. Semantic novelty was considered by contrasting unrelated to related stimuli. Unrelated stimuli can be considered “novel” in that these objects would not normally be grouped together, and are thus semantically novel. Associative novelty was examined during recognition by contrasting objects seen in a new group (rearranged triads) vs. previously seen combinations. In this case, the objects themselves are not novel, this being the third presentation of each object. Instead, it is the grouping of the objects together which is novel, relative to the grouping observed in the first part of the experiment. The unique aspect of this analysis is that these three forms of novelty are very distinct across several perspectives. For example, the stimulus novelty contrast assesses novelty effects from repeated trials which may be separated by several seconds or minutes (with many intervening trials), while semantic novelty effects occur within a single trial (long range vs. short range priming). Therefore, it is not appropriate to focus on what is different in these contrasts as several factors could account for any observed differences. Instead, we focus on the commonalities in activation patterns within these contrasts. If we observe overlapping activity across these very distinct contrasts, this would suggest a possible common neural mechanism for different forms of novelty.

## Participants

Twenty-two participants between the ages of 18–35 were recruited. All participants provided written informed consent and completed a screening questionnaire (for MRI safety and to screen for psychiatric and/or neurological disorders) prior to the experiment. This experiment was conducted in accordance with ethical guidelines at the Montreal Neurological Institute and the Douglas Hospital Research Center, and consistent with the declaration of Helsinki. REB approval at the Montreal Neurological Institute and the Douglas Hospital Research Center was obtained prior to the study.

## Methods

### Experimental task

**Figure 1:**
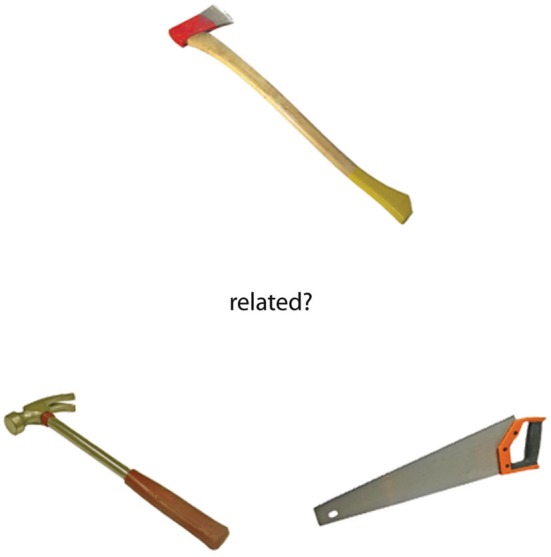
**Example of an encoding trial, presented for 7 s**. Encoding instructions could be either “Related?” or “Smaller?”. The object triad was preceded by the task instruction for 2 s (to serve as a warning that the triad would soon appear), and followed a variable ISI from 1000 to 5000 ms (mean 3000 ms). Trials were identical during the recognition block except the instruction as always “rearranged?”.

Participants performed an episodic memory encoding task followed by a recognition task. It was explicitly stated that this was a memory experiment and that there would be a memory test following the encoding portion, to encourage explicit encoding of the stimuli. During both tasks, participants were shown a task instruction (see below) for 2000 ms, a triad of three objects (one on top and two below) with task instructions for 7000 ms, and finally a fixation cross of variable duration (1000–5000 ms, mean 3000 ms, in 100 ms increments). Images were high quality color photographs of common objects (e.g., tools, office supplies, clothing, dishes, food) from The Bank of Standardized Stimuli (Brodeur et al., 2010). The number of semantic relationships in the triad were modulated, such that there could be no relationships (unrelated triads), one of the two bottom objects could be related to the top object (1-link trials), or all objects could be semantically related (related triads). During encoding, there were two possible task instructions: “related?”, in which case the task was to judge how many of the bottom objects were related to the top object, or “smaller?” in which case the task was to judge how many of the bottom objects were smaller than the top object, in real life (in both cases, resulting in possible responses of 0, 1, or 2). Each encoding instruction was presented for six consecutive trials before switching encoding conditions. There were 16 unique triads for each of the six possible event types. Each triad in the encoding phase was presented twice, with at least six intervening trials between repetitions (mean number of trials between repetitions = 60.4, STD = 40.7, range = 8–154), for a total of 196 events during encoding. An example of an encoding trial is shown in Figure 1.

During the recognition task, half the triads were split into rearranged triads (always using objects shown in the encoding phase, but rearranged into a new configuration), while the other half were left intact. The task instruction was always “rearranged?”, with participants indicating if the triad was intact or rearranged. Each experimental block consisted of 48 trials, and there were a total of four blocks of encoding and two blocks of recognition. Reaction time (RT) data for the encoding task was analyzed using a 2 × 2 repeated measures ANOVA, with the factors novelty (repeated of first presentation) and relatedness (related or unrelated). Recognition RT (intact vs. rearranged) was examined with a paired *t*-test.

### fMRI data acquisition and analysis

Data were collected in a 3T Siemens Tim Trio (TR = 2000 ms, TE = 30 ms, flip angle = 90, 36 slices, 4 mm isotropic voxels, 64 × 64 FOV). Each EPI run of 312 scans was preceded by four excluded scans to allow magnetic steady state. A GLM analysis was performed using SPM8, with images motion corrected, normalized to MNI space and voxels resampled to 2 mm isotropic, and a Gaussian smoothing kernel of 8 mm. The hemodynamic response function (HRF), its first derivative, and dispersion functions were included in the design matrix to improve model estimation, although all contrasts were run on the HRF. Data from three participants had to be excluded for technical reasons (e.g., corrupted imaging data) or below chance performance in the recognition analysis (stemming from pressing the wrong keys on the response pad). In the encoding phase most events in run 1 were first presentation (novel), and most events in run 4 were repeated (second presentation of the triad). As such, these runs were excluded (only the second and third runs of encoding were analyzed). In the recognition phase, only trials in which the participant was correct were included in the statistical contrasts, resulting in the possibility of few events per run of a given event type. In order to maximize the power of the analysis, runs were concatenated (separately for encoding and recognition) by adding an extra regressor for the second run, and a separate linear regressor for each run to remove temporal drift. As a result, a total of 98 events were entered into the first level design matrix. Motion parameters extracted from SPM (3 rotations and 3 translations) were entered into the analysis as regressors of no interest.

We hypothesized that semantically unrelated triads may be considered a form of “novelty”. We constructed two encoding contrasts, one examining “stimulus novelty” (first > second presentation of stimuli, collapsed across relatedness), and the second examining “semantic novelty” (unrelated > related triads, collapsed across repetition). As the 1-link trials have both related and unrelated objects, they were not included in any statistical contrasts to avoid ambiguity (though they were still modeled in the first level GLM during fMRI analysis). Contrasts were also collapsed across encoding instructions. As a result, the stimulus novelty contrast had 28 novel and 36 repeated events, while the semantic relatedness contrast had 32 related and 32 unrelated events. For the recognition data, the contrasts was rearranged > intact, which was considered as “associative novelty” in that the objects presented in each triad are familiar but that specific configuration of objects is novel. In this case, the “novelty” cannot be disentangled from memory effects, as the task was to judge if the triad was “rearranged”. For all contrasts, correction for multiple comparisons (*p* < 0.05 corrected) was performed using a cluster extent threshold determined by monte-carlo simulation (Slotnick et al., 2003), using a voxel threshold of *p* < 0.001 uncorrected. This resulted in an extent threshold of 48 (resampled) voxels, or 384 mm^2^.

Given that the facilitation model posits a relationship between neural responses and reaction times, additional regressors were added to our analysis to identify voxels which were modulated by RT on a trial-by-trial basis (Grinband et al., 2008). This regressor was produced by creating an additional HRF model for each event type with the reaction time (in seconds) of each trial as event duration in SPM. These regressors were then orthogonalized relative to the event HRF model without reaction times and added to the first level (subject) design matrix as additional regressors, along with the standard HRF, derivative, and dispersion. This creates a modulator which is sensitive to changes in HRF. Note that HRF duration reflects a modulation of both amplitude and width of the hemodynamic response, but the regressor is not sensitive to changes which are purely based in amplitude (which will modulate the event-related HRF but not the duration modulator). A main effects analysis collapsing across event types, separately for encoding and recognition and excluding 1-link trials, was then performed to identify voxels in which the *duration* of the HRF was modulated by reaction time on a trial-by-trial basis.

## Results

### Behavioral results

**Figure 2:**
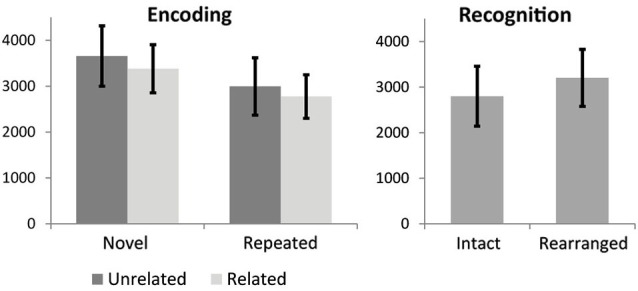
**Reaction time results for encoding conditions**. Error bars show standard deviation.

Repeated measures ANOVA for encoding RTs revealed a main effect of stimulus novelty, MSE = 7.63, *F*_(1,18)_ = 93.6, *p* < 0.000001, with repeated stimuli processed faster than novel stimuli, and a marginal effect of semantic novelty, MSE = 1.18,* F*_(1,18)_ = 3.47, *p* = 0.079, with reduced RT for related triads. No significant interaction was observed. Mean and STD for RTs are shown in Figure 2. Paired *t*-test for recognition RT indicated a significant difference, *t*_(19)_ = −4.4, *p* < 0.0001, with faster RT for intact trials.

### Novelty effects

**Figure 3:**
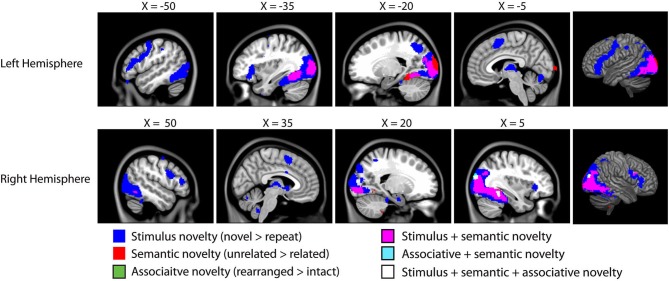
**Results of the fMRI analysis on novelty**. As we are interested in overlap between these distinct forms of novelty, all contrasts are presented on a single brain, with overlapping activity shown in different colors (see legend). All clusters are significant at *p* < 0.05 corrected for cluster extent (48 voxels) at *t* = 3.64 (corresponding to *p* < 0.001 uncorrected). All coordinates in MNI space, images overlaid on the MNI152 template.^1^

**Figure 4:**
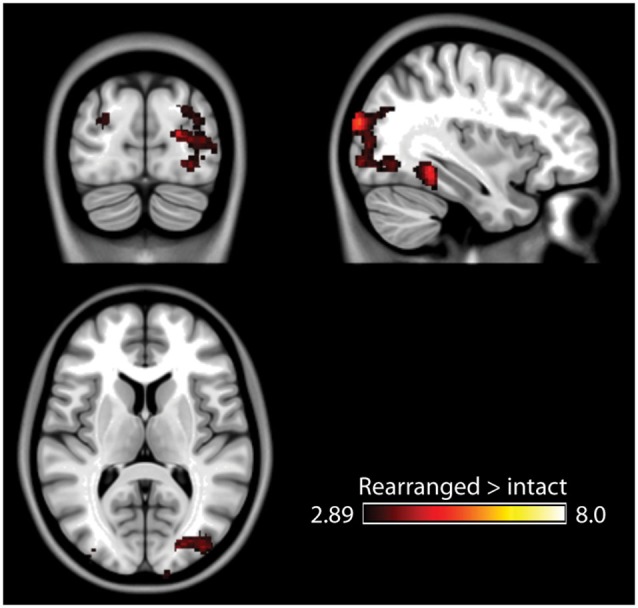
**Results of the associative memory contrast at a more lenient threshold (*p* < 0.005 uncorrected)**. Posterior activity remains spatially constrained relative to the stimulus and semantic novelty contrasts.

The fMRI results of the novelty analysis are presented on Table 1. Figure 3 shows the activations and their overlap across the three novelty contrasts. For both semantic and stimulus novelty, we found large, bilateral activity in the occipital lobes, extending into the superior aspect of the occipital lobe (BA 19), and anterior into the fusiform cortex. For stimulus novelty, a set of activations in the frontal lobes was also observed, including bilateral activation of the inferior frontal gyrus (corresponding to BA 44 and 45, and ventrolateral prefrontal cortex, more extensive on the left), and activity in the medial superior frontal gyrus (BA 6). For semantic novelty, we observed a smaller overlapping activation in the right inferior frontal gyrus (BA 44), and on the left, a more posterior small cluster of activation in the inferior frontal gyrus (BA 6). No overlap with semantic novelty was observed for the stimulus novelty activations in the medial superior frontal cortex, or sub-cortical regions (bilateral external globus pallidus and thalamus). For associative novelty, small clusters on the right were observed overlapping activity in the stimulus novelty contrasts, in BA 19, and the fusiform cortex (BA 37). To ensure that the lack of widespread overlap in associative novelty was not due to thresholding issues, we also examined the associative novelty contrast at a more liberal threshold of *p* < 0.005 uncorrected (Figure 4). While the extent of activity naturally increased, the extensive bilateral posterior activity as observed in semantic and stimulus novelty was still not present for associative novelty even at this liberal statistical threshold.

**Table 1:** **fMRI activations for novelty analysis**.

Voxels (2 mm^3^)	Peak *t*	*X*	*Y*	*Z*	Region
**Stimulus Novelty (novel > repeat)**					
9101	18.75	46	−58	−4	Right occipital, fusiform gyrus
8199	14.17	−42	−74	−8	Left occipital, fusiform gyrus
2382	7.34	−36	32	−12	Left inferior frontal gyrus, BA 47, 45, and 44
773	7.18	8	8	56	Bilateral medial superior frontal gyrus, BA 6
1202	6.89	50	8	28	Right inferior frontal gyrus, BA 45, and 44
402	5.6	−6	−16	6	Left thalamus
158	5.57	26	−56	54	Right superior parietal lobule
145	5.23	8	6	−2	Right caudate/anterior thalamus
60	5.11	6	6	30	Mid-cingulate gyrus, BA 24
57	4.99	66	−2	36	Right superior frontal gyrus, BA 6
54	4.92	10	22	34	Right anterior cingulate (BA 32)
138	4.83	0	−52	−40	Cerebellum midline
85	4.61	−44	−34	44	Left supramarginal gyrus
64	4.2	18	−8	−26	Right hippocampus head
**Semantic Novelty (unrelated > related)**					
3717	9.28	−26	−92	6	Left occipital and fusiform gyrus
3750	8.01	34	−86	−2	Right occipital and fusiform gyrus
90	4.84	40	10	26	Right inferior frontal, BA 44
59	4.66	−56	−8	52	Left premotor cortex (BA6)
**Associative Novelty (rearranged > intact)**					
115	5.58	34	−92	22	Right occipital, BA 18/19
84	5.13	36	−46	−10	Right fusiform gyrus (BA 37)

*XYZ coordinates in MNI space*.

### Trial-by-trial reaction time effects

**Figure 5:**
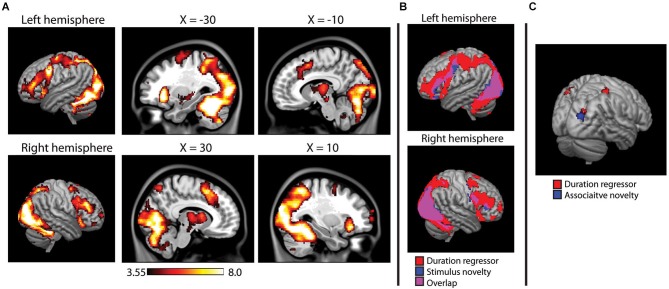
**(A)** Main effects analysis of the HRF duration modulator during encoding, showing voxels in which the duration of the hemodynamic response was modulated by reaction time on a trial-by-trial basis. Activity projected onto a 3D rendering of the cortex (on the MNI152 template), with sagittal slices showing deeper activity. **(B)** Overlap between duration modualtor and stimulus novelty, showing that reaction time modulated the duration on the majority of voxels active in the stimulus novelty contrast. **(C)** Duration modulator during recognition analysis, failing to show the widespread pattern observed at encoding, overlaid on a 3D render of the MNI152 brain. Results of associative novelty contrast also presented to show lack of direct overlap.

The main effects analysis of the duration regressors is shown in Figure 5A. Note that this main effects analysis is independent of the novelty contrasts described above, as it describes voxels in which reaction time affected the duration of the HRF regardless of significant activity in a given voxel. During encoding, the duration regressor was significant across a wide range of regions, including the occipital/fusiform areas observed in the novelty contrasts, superior parietal lobes, prefrontal cortex (middle and inferior frontal gyri) and the thalamus. During the recognition task, however, only three small clusters of activity (right post-central gyrus, left superior parietal, and right superior occipital, BA 19).

Overlap between stimulus novelty and the duration regressor is shown in Figure 5B. The HRF duration was significantly modulated by reaction time in 84.7% of the voxels identified in the stimulus novelty contrast and 94.3% of the voxels identified in the semantic novelty contrast. For associative novelty, no overlap was observed, although one cluster observed in the duration regressor was adjacent to the occipital cluster observed in associative novelty (Figure 5C).

## Discussion

In this study, we performed a data analysis examining the effects of different forms of novelty. While this was not the initial focus of the paradigm, the study design is well fit to an analysis of novelty effects. The key point of this analysis is the distinctions across the different forms of novelty which were assessed. We observed a large posterior activation in response to both stimulus novelty and semantic novelty during episodic memory encoding. A similar pattern has been observed in numerous other studies of stimulus novelty (Henson et al., 2000; Kirchhoff et al., 2000; Koutstaal et al., 2001). The extent of overlap across these distinct contrasts may seem surprising at first, given the number of ways the semantic novelty and stimulus novelty contrasts differ. For example, stimulus novelty occurs across wide-temporal windows, while semantic novelty occurs within a trial. Furthermore, the stimulus novelty contrast differs in response requirements across repeated vs. novel triads, in that when a repeated triad is encountered the given response can be matched to the previous response. Despite the important differences in these contrasts, a large amount of overlap was observed. While numerous papers have shown novelty effects in the posterior cortex, this region of the brain is not typically associated with semantic processing or associative memory. One possible interpretation of these results is that the overlap in neural activity observed in our contrasts is not indicative of processing specifically related to the contrasts of interest, but instead is a secondary consequence of a process common to all three contrasts. When examining behavioral effects, reductions in reaction time were noted in all three conditions (although the effect was only marginal with respect to sematic novelty). The BOLD signal measured in fMRI is linear, such that decreased duration of activity results in decreased amplitude in the signal. We examined this issue with the duration regressor, which showed strong overlap with stimulus and semantic novelty suggesting that the differences in the BOLD response in these voxels were related to changes in the duration of the HRF based on reaction times.

We observed a highly significant reduction in RT for repeated stimuli during encoding, and a trending effect of semantic novelty. A reduction in RT to perform the task may imply less time spent on visual processing of the triads (which were on screen for 7 s, a period well in excess of the average RTs), resulting in less BOLD activity in posterior object processing streams (Goodale and Milner, 1992). This interpretation was born out in the overlap between the duration regressor and the novelty effects, as the HRF duration in almost all voxels observed in the stimulus and semantic novelty contrasts was modulated by reaction time. This suggests a reduction in the duration of neuronal firing during stimulus repetition, most consistent with the facilitation model of repetition suppression which posits a reduction in the duration of neuronal firing without necessarily a change in the number of neurons or rate of firing.

The reduction in neural response to related triads in semantic novelty is likely driven by semantic priming. While in a typical semantic priming paradigm the “prime” is presented prior to the “primed” stimuli, the priming in this case is occurring within a single trial. Most likely, the top object in the triad is serving as the prime, in that the encoding tasks instructed participant to make judgment about the bottom objects relative to the top object in the triads. Thus, the top object served as a semantic prime and facilitates visual processing of the bottom two objects. Semantic priming is known to affect processing time and decrease the magnitude of the BOLD response (Henson, 2003; Henson and Rugg, 2003). For stimulus novelty, any “priming” effects were long range (and thus possibly involving long-term memory), as several intervening trials were presented between repetitions of triads resulting in time ranges from just over a minute to several minutes between novel and repeated stimuli. While it would be inappropriate to focus on differences between stimulus novelty and semantic novelty (as they differ across many factors, including long-range vs. immediate priming effects), the overlapping activity suggests a common mechanism for the reduced neural activity shown for repeated and semantically related stimuli.

In the associative novelty contrast, we did observe some activity in posterior regions in the right hemisphere overlapping the activity seen for stimulus and semantic novelty. However, these regions were substantially constrained in size compared to semantic or stimulus novelty. Further, we did not observe widespread effects of reaction time on the duration of the hemodynamic response. This suggests that while there may be some repetition effects with associative novelty (repeating the combination of objects rather than the actual objects themselves), the effect is much less than stimulus or semantic novelty. But it is important to consider when examining the recognition effects that participants were performing an explicit memory task, which confounds with the “novelty” effects being examined here. As such, the recognition results must be considered with care. Stimulus novelty resulted in the greatest extent of activity (and higher *t*-values). This suggests that stimulus based effects (seeing the images for the first time) has the greatest effect on reducing neuronal firing compared to semantic or associative novelty.

Overall, the results of this study demonstrate a high level of overlap between classic repetition suppression and semantic or associational novelty. This suggests a common mechanism for these findings, which we suggest are priming effects related to neural facilitation. However, we cannot clearly rule out a contribution of other models, such as the sharpening model, which may work together with facilitation to produce the observed results. In addition, while facilitation may fit well with the data presented in this particular paradigm, other models may be appropriate for different paradigms or situations. For example, repeating stimuli multiple times within a short time frame may be best explained via the fatigue model. One important lesson from such a finding is that care must be taken when interpreting the relationship between brain regions and psychological processes. For example, we might be tempted to claim that the posterior region is involved with detecting (or more specifically, rejecting) semantic relatedness, when in fact the observed differences may simply reflect decreases in the duration of neural firing brought out by automatic priming affects and neural facilitation, with the observed regions playing no direct role in semantic evaluation.

## Conflict of interest statement

The authors declare that the research was conducted in the absence of any commercial or financial relationships that could be construed as a potential conflict of interest.

## Footnotes

^1^http://www.bic.mni.mcgill.ca/ServicesAtlases/ICBM152NLin2009
